# Mitral Annular Calcification, a Not So Marginal and Relatively Benign Finding as Many of Us Think: A Review

**DOI:** 10.3390/jcdd12060233

**Published:** 2025-06-18

**Authors:** András Vereckei, Zsigmond Jenei, Hajnalka Vágó, Dorottya Balla, Alexisz Panajotu, Andrea Nagy, Gábor Katona

**Affiliations:** 1 Department of Medicine and Hematology, Semmelweis University, Szentkirályi u. 46, 1088 Budapest, Hungary; 2 Heart and Vascular Center, Semmelweis University, Városmajor u. 68, 1112 Budapest, Hungaryballa.dorottya07@gmail.com (D.B.); panajotu.alexisz@semmelweis.hu (A.P.); horvathne.nagy.andrea@semmelweis.hu (A.N.)

**Keywords:** mitral annular calcification, atrioventricular conduction disturbance, electrocardiography

## Abstract

Mitral annular calcification (MAC) is usually considered an incidental, benign, age-related finding without serious complications in patients evaluated for cardiovascular or pulmonary disease with imaging studies that may result in mitral regurgitation or stenosis when severe. Therefore, it is usually not considered a significant alteration. However, there is accumulating evidence that it is associated with a higher risk of cardiovascular events, such as atherosclerotic coronary artery disease, aortic artery disease, carotid artery disease, peripheral artery disease, stroke, atrial fibrillation, atrioventricular and/or intraventricular conduction disturbance, systemic embolization, infective endocarditis, heart failure and mortality. The presence of MAC also significantly influences the outcome of mitral valve transcatheter and surgical interventions. Several conditions may predispose to MAC. MAC is strongly related to cardiovascular risk factors, such as hypertension, diabetes, smoking and cardiovascular atherosclerosis, and inflammation may also play a role in the pathogenesis of MAC. Also, conditions that increase mitral valve stress, such as hypertension, aortic stenosis and hypertrophic cardiomyopathy, predispose to accelerated degenerative calcification of the mitral annulus area. Congenital disorders, e.g., Marfan syndrome and Hurler syndrome, are also associated with MAC, due to an intrinsic abnormality of the connective tissue composing the annulus.

## 1. Introduction

Mitral annular calcification (MAC) is still considered by many physicians to be a marginal, chronic, degenerative, age-related, benign alteration of the fibrous support of the mitral valve. However, there is accumulating evidence that it can be an active process with features similar to both medial and atherosclerotic cardiovascular calcifications. It is associated with a higher risk of cardiovascular events, such as atherosclerotic coronary artery disease, aortic artery disease, carotid artery disease, peripheral artery disease, stroke, atrial fibrillation, atrioventricular and/or intraventricular conduction disturbance, systemic embolization, infective endocarditis, heart failure and mortality. Its reported prevalence is approximately 10% (8–15%); however, the prevalence of MAC is 42% in elderly patients with known cardiovascular disease, and its prevalence is also significantly greater (40% by echocardiography) in patients with chronic kidney disease [1,2,3,4,5,6,7,8].

## 2. Definition

MAC is defined as the accumulation of calcium along the mitral annulus at the base of the mitral valve, predominantly along its posterior aspect with extension into the posterior leaflet and with increasing severity involvement of the anterior portion of the mitral annulus at the base of the anterior mitral valve leaflet as well [1]. MAC that more commonly affects the posterior than the anterior annulus usually has a C-, J-, U- or O-shape with the open port lying at the site of the aortic outflow tract [9,10]. Although the name suggests confinement of calcification to the mitral annulus, the examination of surgical and autopsy samples revealed extension of calcification into the left ventricle, mitral leaflets, papillary muscle, chordae tendineae and left ventricular outflow tract with occasional continuous calcification of the aorto–mitral continuity extending up into the aortic valve [1,7].

## 3. Diagnosis

**Figure 1:**
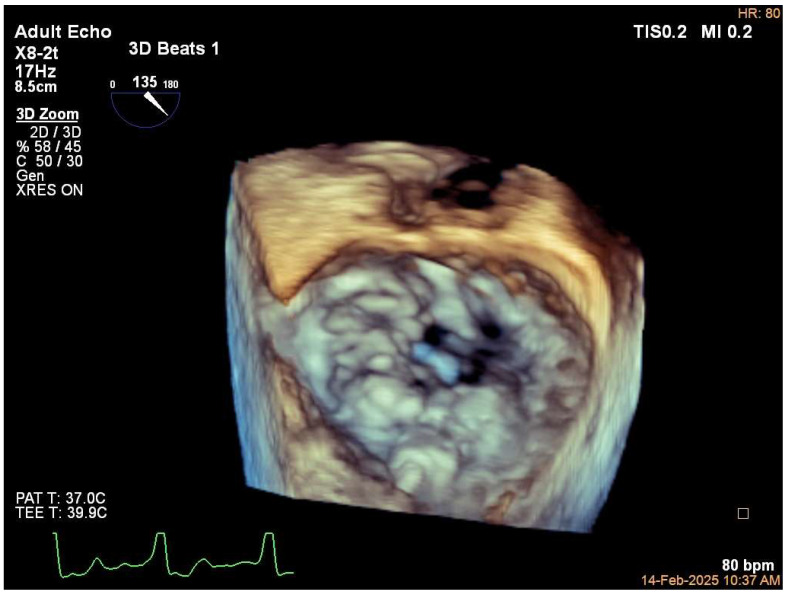
Three-dimensional transesophageal echocardiographic en face “surgical view” visualizing the atrial aspect of the mitral valve in a patient with significant mitral annular calcification (MAC), showing a more pronounced calcification of the posterior mitral annulus.

Upon chest X ray examination, MAC may be visualized as a radiopaque structure following the atrioventricular groove. Previously, echocardiography was considered to be the best method to demonstrate MAC. Its severity was qualitatively determined in the parasternal short axis view at the level of the mitral annulus. Mild severity was characterized by a focal, limited increase in echodensity of the mitral annulus; moderate severity was identified by marked echodensity involving one-third to one-half of the ring circumference; severe MAC was diagnosed when marked echodensity involved one-half of the ring circumference or when intrusion into the left ventricular (LV) inflow tract is revealed. Maximal MAC thickness at its greatest width from the anterior to posterior edge > 4 mm also defined severe MAC. Transesophageal echocardiography provides a more detailed and accurate information about MAC characteristics with its en face “surgical view” of the mitral valve (Figure 1). However, echocardiography is probably not an ideal study for the detection of MAC or valvular calcification because of its relatively low specificity in distinguishing between calcification and dense collagen.

**Figure 2:**
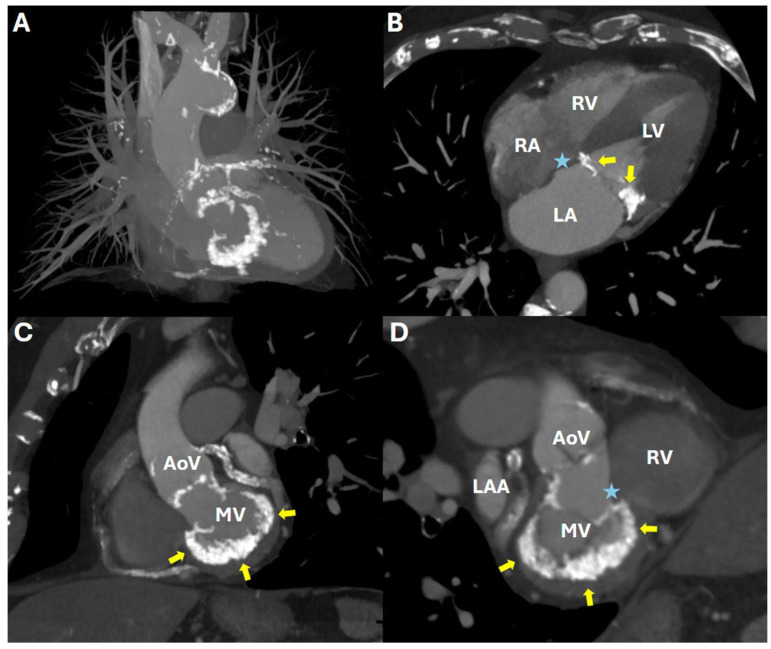
Cardiac computed tomography (CT) images of a patient with significant mitral annular calcification. Panel (**A**): three-dimenstional maximum intensity projection reconstruction overview of the heart and mediastinal great vessels. Panels (**B**,**C**): four-chamber and short axis double oblique reconstructions of the cardiac CT images and Panel (**D**): double oblique reformation of the heart, showing “*en face*” view of mitral annulus, demonstrating a more pronounced posterior and moderate anterior mitral annular calcification (yellow arrows) and the estimated location of the atrioventricular (AV) node (blue asterisks). AoV: aortic valve, LAA: left atrial appendage, LA: left atrium, LV: left ventricle, MV: mitral valve, RA: right atrium, RV: right ventricle.

Cardiac CT is now considered the best method to diagnose and assess the extent and location of MAC (Figure 2) due to its higher spatial resolution and ability to distinguish calcification from fibrous tissue.

**Figure 3:**
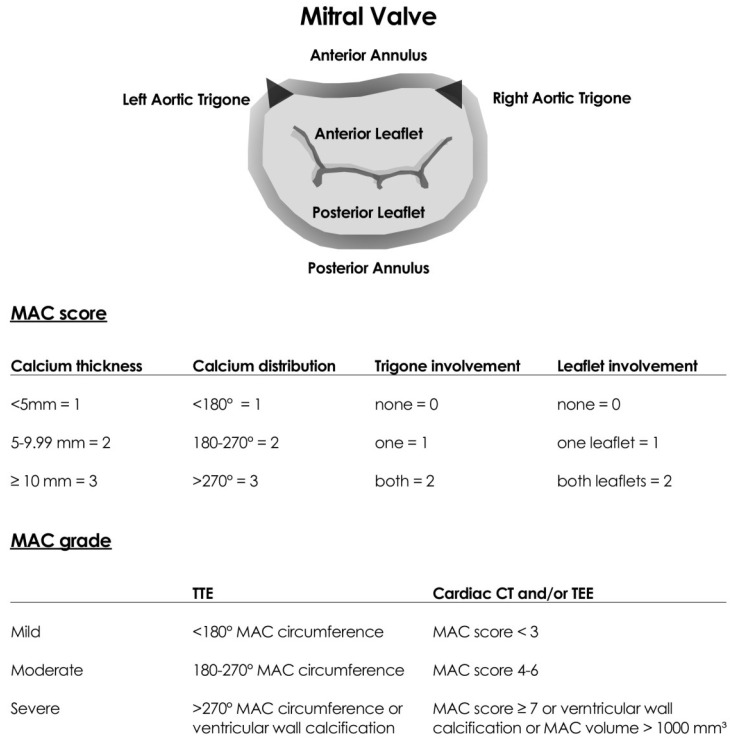
New mitral annular calcification severity grading system by cardiac CT and echocardiography proposed by the Heart Valve Collaboratory working group. CT: computed tomography, MAC: mitral annular calcification, TEE: transesophageal echocardiography, TTE: transthoracic echocardiography. Modified from Table 1 and Figure 1 of Ref. [11].

Recently the Heart Valve Collaboratory working group proposed a new, improved classification of MAC severity grading by applying cardiac CT and/or echocardiography imaging techniques (Figure 3). The MAC severity grading system is based on a MAC score determined by calcium thickness, calcium distribution around the MAC circumference, MAC volume and additional trigone and mitral leaflet involvement [11].

This new grading system is particularly useful when considering the use of devices that require annular or leaflet calcium for anchoring, for surgical considerations and for predicting the risk of embolization. It is also useful for the estimation of the risk for LV outflow tract (LVOT) obstruction, for sealing and paravalvular leak (PVL) in the case of transcatheter mitral valve replacement (TMVR) and for planning mitral valve surgical interventions. Quantification of MAC can also be performed by non-contrast cardiac CT using the calcium score determined with the Agatston method [1,2,6,12,13].

## 4. Pathogenesis

MAC is strongly associated with cardiovascular risk factors, such as hypertension diabetes, obesity, dyslipidemia and smoking. A strong correlation was also demonstrated between MAC and aortic atheroma, carotid atherosclerotic disease, peripheral artery disease and coronary artery disease. These observations support the hypothesis that the pathogenesis of MAC and atherosclerosis are strongly associated [2,3,6,14]. In contrast to the risk of incident coronary artery and aortic valve calcification, which was lower in women than in men, female gender was a risk factor for MAC [1,14], whereas the prevalence of calcification in the descending thoracic aorta was greater in women [14]. These findings suggest a gender-related pathophysiologic process affecting the calcification at different anatomical sites, which might be related to the paradoxical relationship between bone and cardiovascular mineralization. MAC usually develops later in life than coronary artery and aortic valve calcification and might be related to age-related osteoporosis affecting mainly women, in which severe bone loss can be associated with ectopic calcium deposits that may cause MAC [14,15]. Another important difference is that genetic predisposition to elevated LDL-cholesterol was associated with aortic valve calcification and incidence of aortic valve stenosis, which was not associated with MAC, but genetic disposition to elevated triglyceride levels was associated with the presence of MAC but not with aortic valve calcification and aortic valve stenosis [1,16,17].

Also, conditions that increase mitral valve stress, such as hypertension, LV hypertrophy, mitral valve prolapse, aortic stenosis and hypertrophic cardiomyopathy, are associated with accelerated degenerative calcification of the mitral annulus area [1,2,9,18]. Connective tissue disorders, e.g., Marfan syndrome, are also associated with MAC, which may be due to increased mitral stress caused by associated mitral valve prolapse with Marfan syndrome or due to an intrinsic abnormality of the connective tissue composing the annulus. MAC has been reported in children with Hurler syndrome as well. Abnormal fibroblasts and accelerated collagen degeneration may be responsible for the early appearance of MAC in these patients [1,2,9,18]. Inflammation may also play a role in the pathogenesis of MAC. C-reactive protein (CRP) was identified to have an independent association with MAC, and interleukin-6 was identified as a risk factor for MAC. GlycA, a novel composite serum biomarker for systemic inflammation reflecting the serum level and glycosylation of acute serum reactants, has been found to be associated with incident MAC and MAC progression. ^18^F-Fluorodeoxyglucose (FDG) positron emission tomography (PET) uptake was also observed in MAC as a marker of inflammation [1,8,14]. The common occurrence of MAC in patients with chronic renal failure is probably related to abnormal calcium-phosphorus metabolism. Due to associated secondary hyperparathyroidism, the calcium-phosphorus product is elevated and exceeds its solubility in serum leading to tissue deposition (metastatic calcification) [2,7].

## 5. Clinical Implications

Although MAC is still an incidental and asymptomatic finding in some patients, its clinical significance is that, in addition to its potential role in causing mitral valve dysfunction, it is associated with a higher risk of cardiovascular disease and mortality, and in the presence of some cardiovascular events one should think of its etiological role. Before discussing in detail its clinical implications, we illustrate this with a typical interesting case.

### 5.1. Case

A 72-year-old symptomless male patient presented at our department for cardiovascular screening examination in April 2022. In his past history, hypercholesterolemia was known from 2010, which was sometimes associated with mildly elevated, sometimes with normal triglyceride levels, indicating that the occasional mild hypertriglyceridemia was due to dietary causes. He was not aware of hypertension. His thyroid function and CRP level were repeatedly normal. He was in a good physical condition and could well tolerate physical exercise and played tennis regularly. He did not have chest pain or shortness of breath either in resting state or during physical exercise. His blood pressure was 145/85 mmHg on both arms; heart rate was 72–78 bpm; his heart was enlarged 0.5–1.0 cm to the left by percussion; a grade 1/6 ejection type systolic murmur was heard over all auscultation areas. A decreased alveolar breathing sound was heard over the lungs. No other alterations were found with the physical examination.

**Figure 4:**
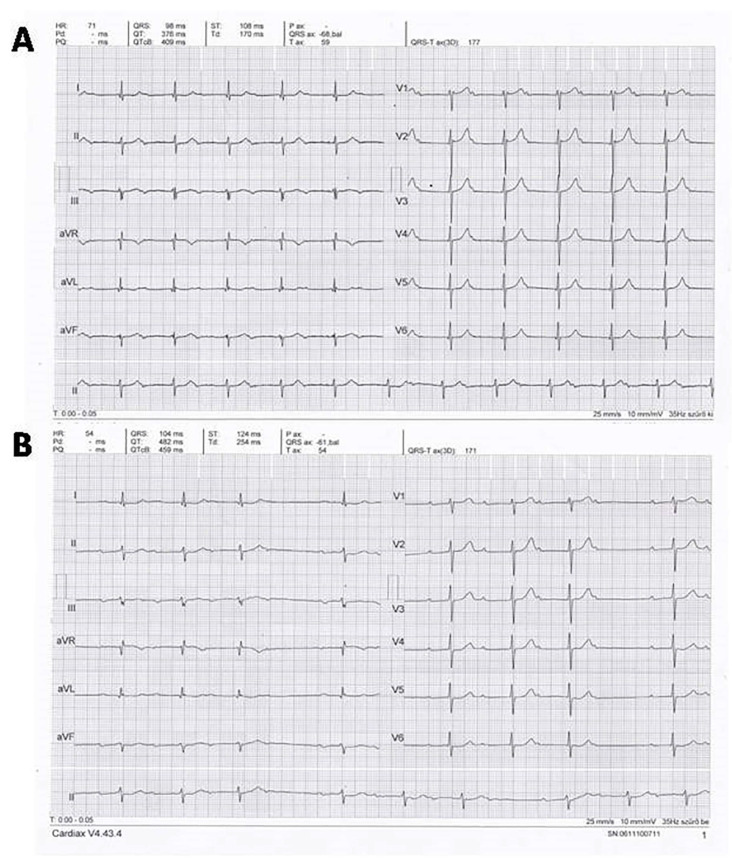
ECGs recorded at the initial presentation. Panel (**A**) (initial ECG): 68 bpm sinus rhythm, significant left axis deviation with a frontal plane QRS axis of approximately −40°, PR interval: 520 ms [first-degree atrioventricular (AV) block]. Fragmented QRS complexes are present in leads III, aVL and aVF, positive–negative P waves are seen in the inferior leads corresponding to third-degree interatrial block predisposing to atrial fibrillation. Mildly elevated ST segments in leads V_1–2_. Panel (**B**): ECG recorded 24 days after the initial presentation showing Mobitz type I second-degree AV block (AV Wenckebach periodicity) with blocked, non-conducted P waves. The PR interval of the first QRS complex after the pause was still significantly prolonged: 360 ms. Otherwise, the ECG was identical to the initial ECG.

During the initial examination, the ECG (Figure 4A) revealed a 68 bpm sinus rhythm with significant (−40°) left axis deviation, fragmented QRS complexes in leads III, aVL, aVF, first-degree AV block with extremely prolonged PR interval (PR = 520 ms). There were positive–negative P waves in the inferior leads suggesting third-degree interatrial block. An ECG recorded 24 days later in May 2022 (Figure 4B) showed sinus rhythm with second-degree Mobitz Type I AV block with a 54 bpm average ventricular rate; otherwise, it was identical to the previous ECG. The PR intervals, even immediately after the pauses, were significantly prolonged (360 ms).

**Figure 5:**
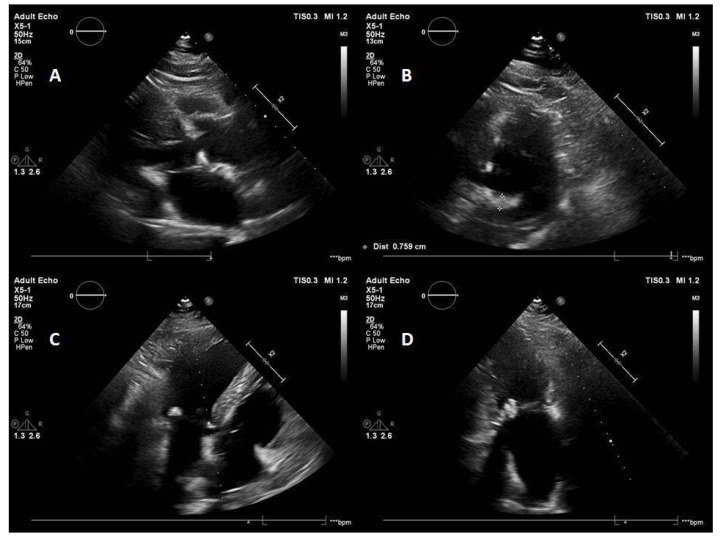
Echocardiographic examination of the patient. Panels (**A**–**D**): parasternal long axis and short axis, apical 4-chamber and 2-chamber views showing the conspicuous posterior and mild anterior mitral annular calcification and mild calcification and thickening of the aortic valve. Panel (**B**): parasternal short axis view at the level of mitral annulus reveals a posterior mitral annular calcification of 8 mm, which exceeds the significant level (>4 mm).

The initial echocardiography (Figure 5) revealed a minimally thickened and calcified tricuspid aortic valve, otherwise normal heart valves, mitral annulus calcification with a significant (of >4 mm thickness) [9,10] posterior and milder anterior annulus involvement, minimal concentric LV hypertrophy, minimally-to-moderately dilated aortic root and ascending aorta and lipomatous atrial septum hypertrophy. The systolic LV and right ventricular (RV) function was normal; relaxation abnormality was present. Grade I mitral regurgitation, slight-to-grade I aortic regurgitation, slight tricuspid and pulmonary regurgitations and slight mitral stenosis were seen. The underlying cause of mild mitral regurgitation and slight mitral stenosis was the mitral annulus calcification. The estimated pulmonary artery systolic pressure was 23 mmHg. Chest X ray was completely negative. In May 2022, a Holter examination revealed sinus rhythm with first-degree AV block and sometimes second-degree Mobitz type I AV block. The minimal heart rate was 37 bpm at night; there were five pauses > 2400 ms, and the longest pause was 2609 ms. The patient did not have any complaint during the ambulatory ECG monitoring. A mild to moderate hypertension was confirmed by home blood pressure measurements immediately after the initial examination, which was treated with 150 mg/day irbesartan. The detailed routine laboratory examination revealed moderate hypercholesterolemia and mildly increased serum triglyceride level (1.9 mmol/L), a mildly increased NT-pro-BNP (234 pg/mL, normal value < 125 pg/mL), and, therefore, an additional 10 mg/day rosuvastatin therapy was recommended.

**Figure 6:**
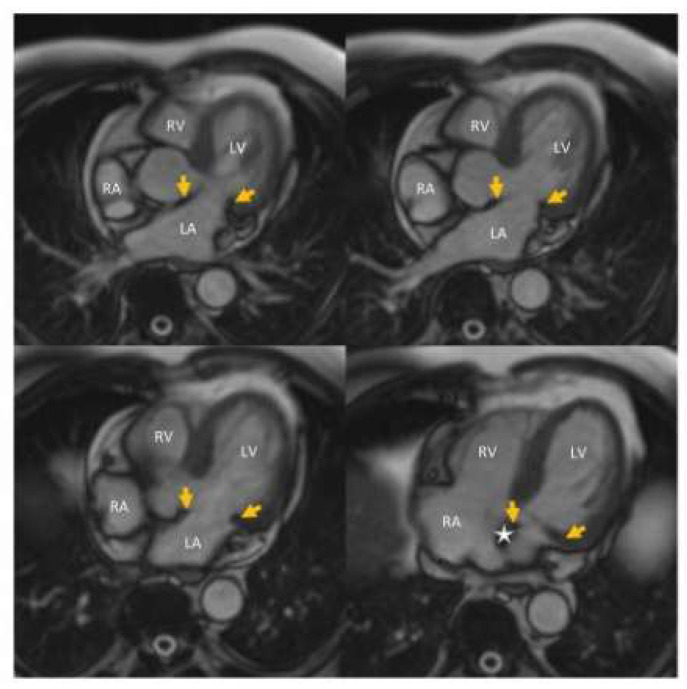
Cine movie cardiac MR images in transversal planes. Arrows show the mitral annular calcification. Suspected area of the AV node is indicated by asterisk, showing its proximity to MAC.

**Figure 7:**
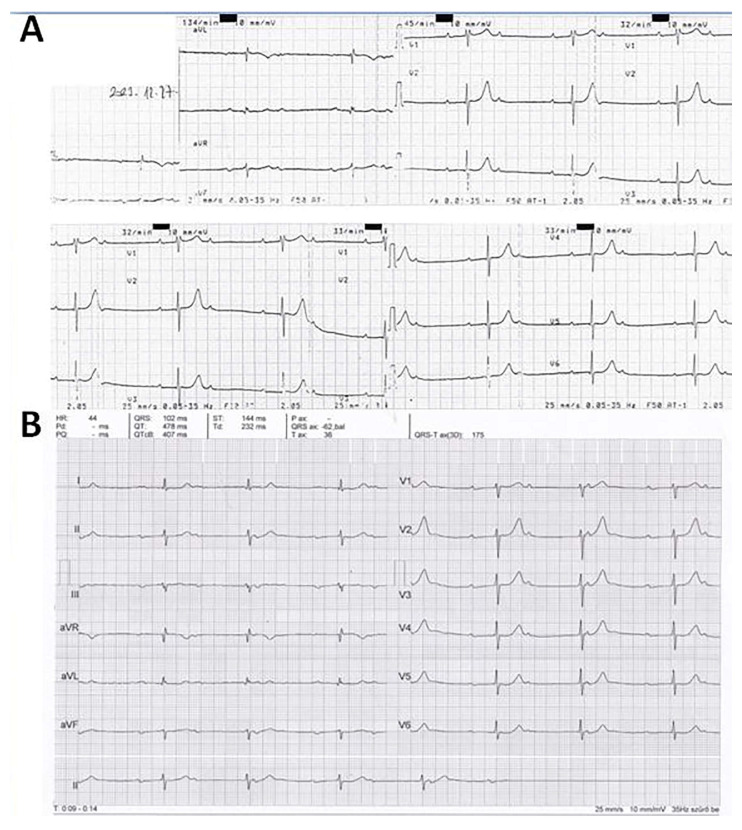
ECGs recorded 18 months after the initial presentation before pacemaker implantation. Panel (**A**): sinus rhythm with second-degree Mobitz type I AV block with 2:1 AV conduction and a 34 bpm ventricular rate. Panel (**B**): sinus rhythm with second-degree Mobitz type I AV block with 3:2 AV conduction and a 44 bpm ventricular rate.

After the initial work-up, the underlying cause of the first-degree AV block with extreme PR interval prolongation and 1:1 AV conduction alternating with second-degree Mobitz type I AV block was not clear, the verified mild-to-moderate hypertension and hypercholesterolemia did not seem a sufficient explanation. We did not perform coronary CT angiography examination because the patient did not have any complaint suggesting even the mild suspicion of coronary artery disease. Looking for the potential explanation, we realized that mitral annulus calcification revealed by echocardiography can be associated with AV and intraventricular conduction disturbance due to its location very close to the AV node and the bundle of His. He underwent a cardiac MRI examination in October 2022 in order to rule out or confirm any other potential underlying cause of the AV block and check the potential atrial abnormalities that may cause third-degree interatrial block. Cardiac MRI examination revealed normal LV and RV systolic function, normal-sized cardiac chambers, moderate LV hypertrophy, otherwise normal heart. The left atrial size was also normal, and due to the thin atrial wall, the presence or absence of atrial fibrosis could not be properly assessed. Cardiac MRI examination also showed the mitral annulus calcification with posterior predominance and close location to the AV node and bundle of His (Figure 6). The same clinical and ECG presentation and lack of symptoms persisted until December 2023. On 27 December 2023, he detected a very low pulse rate of 25–45 bpm with his automatic sphygmomanometer while he was still symptomless. The ECG (Figure 7A,B) showed a second-degree Mobitz type I AV block with 2:1 and 3:2 AV conduction, with a 34–44 bpm ventricular rate. Due to the progression of AV block and its association with significant bradycardia, an urgent DDD pacemaker implantation was performed.

This brief case report is an example that the usual consideration of MAC as a marginal, asymptomatic, age-related, degenerative, benign alteration, that may not cause any complication and is, therefore, irrelevant, or may result in mitral regurgitation or mitral stenosis when more severe, is not correct in a non-negligible number of cases.

### 5.2. Mitral Valve Dysfunction

MAC most commonly leads to mitral regurgitation, which results from calcium infiltration of the base of the posterior leaflet, reducing leaflet mobility, elevating the leaflets and, thus, facilitating chordal elongation or rupture and distorting leaflet coaptation. An alternative mechanism of mitral regurgitation is a MAC-related compromise of the sphincter-like function of the mitral annulus during systole [19,20].

Mitral stenosis due to MAC is quite rare and, unlike rheumatic mitral stenosis, there is a sparing of the leaflet commissures in these patients. MAC causes mitral stenosis by encroaching on mitral orifice area or by restricting anterior leaflet motion. Rheumatic mitral stenosis is associated with commissural fusion, leaflet tip restriction and chordal shortening with relative sparing of the annulus and the base of mitral leaflets. The limiting orifice area is at the rheumatic leaflet tips, whereas in mitral stenosis, due to MAC, the limiting orifice area is at the base of the mitral leaflets. The non-rheumatic, degenerative mitral valve stenosis is largely related to MAC; its proportion among mitral stenosis cases was 12.5%, which increases significantly with age [1,2,20].

In patients with hypertrophic obstructive cardiomyopathy, especially in older patients, MAC may contribute to systolic anterior motion, anterior position of the mitral valve as well, causing functional LV outflow tract obstruction [21].

### 5.3. Other Cardiovascular Disease, Mortality

Evidence is accumulating that, in addition to mitral valve disease, MAC is associated with a higher risk of cardiovascular events and mortality. A strong association of MAC was shown with cardiovascular atherosclerosis, such as aortic atheroma, carotid atherosclerotic disease, peripheral artery disease, coronary artery disease, and it may be associated with an increased risk of stroke [1,2,4,12,22]. MAC may predispose patients to systemic embolization by its association with complex aortic atheroma, a high-risk emboligenic substrate, by providing calcium spicules, which may embolize, or providing a nidus for platelet and fibrin deposition [1,9,23]. Severe MAC can cause caseous necrosis of the mitral valve, a tumor-like mass-occupying lesion, having a toothpaste-like content, thought to be the product of liquefactive necrosis [1,24]. Patients with MAC have an increased prevalence of AV block and/or intraventricular conduction disturbances [1,2,3,10,18,25,26], as the example of our patient demonstrated. MAC also predisposes to increased atrial fibrillation incidence that may be due to left atrial enlargement and/or interruption of inter- or intra-atrial conduction [1,2,9,18,27,28]. Although atrial fibrillation did not occur yet, an increased risk of atrial fibrillation is also present in our patient as indicated by the presence of a third-degree interatrial block on his ECG. In the Cardiovascular Health study, MAC was also independently associated with congestive heart failure, increasing its risk by 70%, whereas a more than mild (moderate or severe) MAC increased its risk by 140% [12]. MAC may also serve as a nidus for infective vegetation, which may be due to endocardial inflammation and disruption of endothelial surface or even ulceration associated with annular calcium and, therefore, may predispose subjects to infective endocarditis, especially caused by Staphylococcus aureus [29]. MAC was also associated with reduced bone mineral density, dementia and, particularly in a high burden of MAC, with increased risk of renal failure. In addition, MAC is associated with increased cardiovascular, all-cause and noncardiovascular mortality. Thus, a reverse association with chronic renal failure is also true; not just patients with chronic renal failure, particularly those undergoing hemodialysis, are predisposed to MAC, but the presence of MAC is associated with an increased risk of incident renal failure [8].

### 5.4. The Effect of MAC on Transcatheter and Surgical Cardiac Interventions

We summarize briefly the influence of MAC on these interventions, but the detailed overview of this topic and these interventions is beyond the scope of this review.

In patients with aortic valve stenosis, undergoing transcatheter aortic valve replacement (TAVR) outcomes are worse in patients with MAC [30]. The presence of MAC doubles the risk of all-cause mortality and nearly triples the odds of permanent pacemaker implantation after TAVR. The degree of MAC severity by CT calcium score is an independent predictor of conduction system disturbances (left bundle branch block or high-degree AV block) after TAVR. Mitral regurgitation, which is frequently present in patients with calcific aortic stenosis, usually improves significantly after TAVR, but MAC with calcification encroaching onto the leaflets and restrictive leaflet motion were associated with reduction of mitral regurgitation improvement [30,31,32,33]. Manipulations of wires and large balloons during TAVR may increase calcium debris, resulting in brain or coronary emboli in patients with severe MAC [2].

The two main treatments of mitral valve dysfunction associated with MAC are surgical and transcatheter interventions. Usually, mild posterior annulus MAC affecting less than one-third of the annular circumference does not affect surgical valve replacement or repair using conventional techniques [13]. The most effective and beneficial surgical approach to patients with mitral valve disease associated with severe MAC is the deep debridement of annular calcium with resection of the affected segments followed by reconstruction. The reconstruction is performed by using annular suturing, an atrial flap or an autologous pericardial patch. This technique is followed by mitral valve repair, if possible, or by implantation of a prosthetic valve [11,19,34]. When deep surgical debridement of MAC is performed, it may be a contributing factor to complications, such as cardiac rupture at the AV junction, rupture of the LV free wall and injury to the circumflex artery [1,2,19,34]. However, because of the complexity of the operation and the potential catastrophic complications, this operation can be performed by very experienced surgeons in heart valve surgery centers. Therefore, a contemporary surgical practice favors targeted decalcification using ultrasonic debridement, which allows focal decalcification without fracturing the calcium embedded into the ventricular wall, rendering the option of working around MAC, where possible. However, the standard deep debridement surgical procedure seems to provide the most durable solution [11,13,19,34]. A hybrid approach consisting of surgical valve in MAC (ViMAC) insertion of a balloon-expandable transcatheter heart valve (THV) via left atriotomy and deployment under direct vision can avoid the need of annular decalcification in high-risk surgical patients with severe and circumferential MAC. Additional atrial sutures prevent migration, and supplementary pericardial/Teflon felt strips can be used to decrease paravalvular leaks. Resection of the anterior mitral valve leaflet can also be performed via direct atrial approach to reduce the risk of LVOT obstruction. Although this approach is a good alternative in high-surgical-risk and comorbid patients, its longer term mortality at 1 year is >30% [13,34,35]. Patients with extensive calcium extending to the aortomitral curtain and an intercommissural distance > 35 mm may not be suitable for the transatrial surgical approach. An intercommissural distance > 35 mm may be too large for a SAPIEN family valve; an exuberant overexpansion can result in catastrophic atrioventricular groove injury or significant transvalvular regurgitation [11]. The surgical outcome in patients with mitral valve disease associated with significant MAC are inferior compared to those without significant MAC [11,13].

Percutaneous balloon valvuloplasty is not recommended in patients with degenerative mitral valve stenosis due to MAC, since this is not an ideal treatment because of the absence of commissural fusion and because the narrowest valve area is usually at the mitral inflow orifice in these patients [11].

Transcatheter edge-to-edge repair (TEER) technique with MitraClip or PASCAL devices is considered first in high-surgical-risk patients with MAC and either primary or secondary severe mitral regurgitation (MR). TEER is usually not recommended in patients with severe MAC with mitral stenosis, with a mitral orifice area < 3 cm^2^ and/or a mitral inflow mean gradient > 5 mmHg, with calcium extension into the mitral leaflets, and/or with restricted leaflet motion or with a short and restricted posterior mitral leaflet. However, two case series of 28 and 61 patients with moderate to severe MAC undergoing TEER did not find differences in procedural success when compared with patients without MAC. In one of these studies, the 1-year mortality tended to be higher in patients with MAC (19.7% vs. 11.3%, *p* = 0.05) [35,36,37,38].

Symptomatic high-surgical-risk patients with MAC and severe MR who are likely to develop severe mitral stenosis with TEER can be considered for transcatheter mitral valve replacement (TMVR). Transseptal ViMAC using the SAPIEN family aortic transcatheter heart valve (THV) can be performed in the presence of severe MAC (MAC score ≥ 7), which is necessary for anchoring this balloon-expandable valve. However, in patients with MAC, circumferential calcification is not commonly present. Patients with a MAC score ≤ 6 have a very high risk of device migration or embolization. The most important complications of transseptal SAPIEN family aortic THVs are device migration or embolization and LVOT obstruction or paravalvular leak. In most high-surgical-risk patients with MAC and severe MR, who are unsuitable for TEER, TMVR with dedicated mitral THVs, such as the Tendyne, Intrepid and SAPIEN M3 valves, can be tried because these devices do not require circumferential calcification for anchoring/fixation. They have other features for anchoring, such as anatomical shape, fixation barbs, a “champagne cork” valve conformation, an apical tether or the creation of an “anchoring dock” by utilizing the native mitral leaflets in the case of SAPIEN M3 system. The SAPIEN M3 mitral THV is a modified form of SAPIEN 3 aortic THV; it is also a balloon-expandable valve, but it has a coiling nitinol docking system, providing it a dedicated shape. Its acute technical success rate was 86%, and 12.1% of patients had ≥ moderate MR. The other dedicated self-expanding mitral THVs are the Tendyne valve, which is implanted transapically, and the Intrepid valve, which can be implanted either transapically or transseptally. These valves had higher acute technical success rates (97% and 94%, respectively), and a greater percent of these patients have no or trivial MR (93.2% and 96%, respectively, and the rest of the patients had only mild MR) compared with the SAPIEN M3 mitral THV. TMVR with dedicated valves achieved a more effective MR reduction than TEER (<1 + MR 95.8% vs. 68.8%, *p* < 0.001). Asymmetric calcification of the mitral annulus in patients with TMVR may result in significant paravalvular regurgitation, which is generally poorly tolerated. Severe MAC may also predispose to annular rupture during valve deployment. Significant posterior annular calcification (which may push the valve anteriorly) may predispose to LVOT obstruction [11,39,40,41]. Cardiac CT is crucial in the preoperative evaluation of patients with MAC who are either candidates for cardiac surgery or transcatheter procedures, in defining anatomical eligibility, predicting the risk for adverse outcomes (e.g., device embolization) and calculating the “neo LVOT”, the minimal LVOT area that is expected after transcatheter valve deployment. A neo LVOT area < 1.8 cm^2^ is a reliable predictor of the risk for postprocedural LVOT obstruction, a common and serious complication of ViMAC procedures [11,42].

### 5.5. Treatment

Currently no confirmed, well-established medical therapy is available for MAC. Therefore, medical treatment focuses on the treatment of risk factors, comorbidities and symptoms. Other potential future therapies may be triglyceride lowering and anti-inflammatory therapies, since inflammation and hypetriglyceridemia may play a role in the pathogenesis of MAC. Fibroblast growth factor-23 (FGF-23), which, by increasing renal phosphate excretion and inhibiting 1,25-dihydroxyvitamin D synthesis, increases the risk for vascular calcification, was positively associated with MAC incidence and progression. Therefore calcium, or a calcimimetic agent, such as cinacalcet, that lowers FGF-23 level, may be a potential treatment for MAC [1,35,43,44]. Fetuin A, a glycoprotein synthesized in the liver, which binds calcium and phosphate, is a circulating inhibitor of vascular calcification, and its level was inversely associated with MAC. Omega-3 fatty acids might be a potential therapy for MAC in the future, acting by increasing fetuin A and decreasing triglyceride levels in the serum [44,45].

## 6. Conclusions

When we have a patient evaluated for the suspicion of cardiovascular or pulmonary diseases with imaging studies which reveal MAC, it can be still a marginal, insignificant alteration in many cases. However, since there is accumulating evidence that MAC is associated with an increased risk of many cardiovascular complications and mortality, which we discussed in detail in this review, when any of these cardiovascular complications is present in the patient, the attending physician should always consider whether MAC is the potential underlying cause. Furthermore, the influence of a significant MAC should always be considered on certain planned surgical or transcatheter interventions.

## Data Availability

Not applicable.
